# Reflexões sobre as Taxas de Sobrevida em Pacientes com Fenocópia de Brugada

**DOI:** 10.36660/abc.20250330

**Published:** 2025-10-28

**Authors:** Gabriel Scarpioni Barbosa, José Nunes de Alencar, Adrian Baranchuk

**Affiliations:** 1 Faculdade Santa Marcelina Divisão de Medicina São Paulo SP Brasil Divisão de Medicina – Faculdade Santa Marcelina, São Paulo, SP – Brasil; 2 Instituto Dante Pazzanese de Cardiologia Divisão de Pesquisa São Paulo SP Brasil Divisão de Pesquisa – Instituto Dante Pazzanese de Cardiologia, São Paulo, SP – Brasil; 3 Queen's University Divisão de Cardiologia – Kingston Health Science Centre Ontário Canadá Divisão de Cardiologia – Kingston Health Science Centre, Queen's University, Ontário – Canadá

**Keywords:** Síndrome de Brugada, Taxa de Sobrevida, Eletrocardiografia

Lemos com grande interesse o artigo "Sobrevida em Pacientes com Fenocópia de Brugada. Série de Casos" de Fonseca et al.^1^ Os autores descrevem uma série de casos de pacientes com Fenocópia de Brugada (FB) e relatam a sobrevida hospitalar. Elogiamos os autores por abordarem um tópico importante relacionado à FB; no entanto, gostaríamos de comentar as considerações metodológicas e propor recomendações que possam fortalecer a interpretação e orientar futuros relatos sobre FB.

FB se refere a condições clínicas que levam ao padrão de Brugada no ECG (PEBr) sem o substrato genético de Síndrome de Brugada (SB) e uma condição subjacente que justifique a alteração do ECG.^2^ O termo foi usado pela primeira vez para descrever o PEBr causado pela síndrome de infusão de propofol; no entanto, após várias correções ao conceito, os medicamentos que bloqueiam os canais de sódio não foram mais considerados parte do espectro da FB.^3^ A FB representa um desafio diagnóstico mesmo para especialistas. A precisão média para identificar FB com base apenas nos ECGs de 12 derivações foi de 43±33% entre 10 especialistas internacionais.^4^ Portanto, o contexto clínico é essencial para diferenciar FB de SB. Anselm et al.^5^ apresentaram um critério diagnóstico sistemático (Tabela 1) para o diagnóstico de FB. A ênfase foi colocada no reconhecimento da condição clínica subjacente e na baixa probabilidade pré-teste para FB, além da resolução do PEBr imediatamente após a resolução da condição subjacente. Com base na descrição limitada fornecida por Fonseca et al.,^1^ não podemos excluir a possibilidade de que alguns casos representassem SB oculto, desmascarado por hipertermia ou outros gatilhos. Esse viés de classificação incorreta poderia, por si só, distorcer a mortalidade observada.

**Tabela 1:** Critérios diagnósticos sistemáticos para a fenocópia de Brugada

I) O padrão de ECG tem características morfológicas de Brugada tipo 1 ou tipo 2. (Obrigatório).
II) O paciente tem uma condição subjacente identificável. (Obrigatório).
III) O padrão do ECG desaparece após a resolução da condição subjacente. (Obrigatório).
IV) Há uma baixa probabilidade pré-teste clínico de síndrome de Brugada verdadeira, determinada pela ausência de sintomas, histórico médico e histórico familiar. (Obrigatório).
V) Resultados negativos em testes provocativos com bloqueadores dos canais de sódio, como ajmalina, flecainida ou procainamida.
VI) O teste provocativo não é obrigatório se a manipulação cirúrgica do trato de saída do ventrículo direito tiver ocorrido nas últimas 96 horas.
VII) Os resultados dos testes genéticos são negativos (desejável, mas não obrigatório, porque a mutação SCN5A é identificada em apenas 20% a 30% dos probandos afetados pela verdadeira síndrome de Brugada).

As causas da FB são diversas, e o conhecimento dessas associações é importante para o diagnóstico correto. O PEBr foi associado a doenças vasculares oclusão coronariana aguda (OCA)^6^ e embolia pulmonar aguda),^7^ distúrbios hidroeletrolíticos (hipocalemia^8^ e hipercalemia),^9^ variações anatômicas (pectus excavatum)^10^ e outras condições clínicas (choque séptico^1^ e pericardite aguda).^11^

Dado que a FB não possui o substrato patológico da SB, sua mortalidade reflete inerentemente a gravidade da condição subjacente. A SB possui um mecanismo de evolução e taxa de sobrevida bem estabelecidos. Pacientes com SB são propensos a arritmias ventriculares, que podem levar à morte cardíaca súbita. A mortalidade relatada é de aproximadamente 1,7% em um período médio de acompanhamento de 73,2 ± 58,9 meses.^12^ Fonseca et al.^1^ relatam um estudo de taxa de sobrevida hospitalar de 26,8%, mas esse número é fortemente influenciado pela predominância de condições graves na coorte, como OCA, embolia pulmonar aguda e choque séptico. Se esta série tivesse incluído pacientes ambulatoriais ou quadros mais leves, as estimativas de sobrevida provavelmente seriam substancialmente maiores, refletindo viés de encaminhamento.

Para avaliar com mais precisão o prognóstico da FB, investigações futuras devem considerar o diagnóstico diferencial, incluindo a própria SB, e inscrever coortes maiores e mais heterogêneas, e comparar os resultados em pacientes com condições subjacentes idênticas que se apresentam com PEBr versus aqueles com padrões de ECG típicos (por exemplo, OCA com PEBr versus OCA com supradesnivelamento de ST convencional).

Reconhecemos que a série de casos de Fonseca et al.^1^ contribui com dados preliminares valiosos sobre o prognóstico da FB. No entanto, uma avaliação crítica de potenciais vieses — incluindo encaminhamento, classificação incorreta e fatores de confusão — é essencial para delinear a verdadeira aplicabilidade e generalização dos achados

## References

[1] Fonseca LMT, Cedeño RA, Bello LAH, Díaz-Heredia J, Quezada DMP, Castro RAG (2025). Survival in Patients with Brugada Phenocopy. Case Series. Arq Bras Cardiol.

[2] Baranchuk A, Nguyen T, Ryu MH, Femenía F, Zareba W, Wilde AA (2012). Brugada Phenocopy: New Terminology and Proposed Classification. Ann Noninvasive Electrocardiol.

[3] Riera AR, Uchida AH, Schapachnik E, Dubner S, Ferreira C, Ferreira C (2010). Propofol Infusion Syndrome and Brugada Syndrome Electrocardiographic Phenocopy. Cardiol J.

[4] Gottschalk BH, Anselm DD, Brugada J, Brugada P, Wilde AA, Chiale PA (2016). Expert Cardiologists Cannot Distinguish between Brugada Phenocopy and Brugada Syndrome Electrocardiogram Patterns. Europace.

[5] Anselm DD, Gottschalk BH, Baranchuk A (2014). Brugada Phenocopies: Consideration of Morphologic Criteria and Early Findings from an International Registry. Can J Cardiol.

[6] Ferrando-Castagnetto F, Garibaldi-Remuñan A, Vignolo G, Ricca-Mallada R, Baranchuk A (2016). Brugada Phenocopy as a Dynamic Electrocardiographic Pattern during Acute Anterior Myocardial Infarction. Ann Noninvasive Electrocardiol.

[7] Zhan ZQ, Wang CQ, Nikus KC, Pérez-Riera AR, Baranchuk A (2014). Brugada Phenocopy in Acute Pulmonary Embolism. Int J Cardiol.

[8] Genaro NR, Anselm DD, Cervino N, Estevez AO, Perona C, Villamil AM (2014). Brugada Phenocopy Clinical Reproducibility Demonstrated by Recurrent Hypokalemia. Ann Noninvasive Electrocardiol.

[9] Dahal K, Shrestha D, Hada R, Baral A, Sherpa K (2020). Hyperkalemia Mimicking Brugada Pattern in Electrocardiogram: A Rare Case Report from Nepal. Saudi J Kidney Dis Transpl.

[10] Awad SF, Barbosa-Barros R, Belem LS, Cavalcante CP, Riera AR, Garcia-Niebla J (2013). Brugada Phenocopy in a Patient with Pectus Excavatum: Systematic Review of the ECG Manifestations Associated with Pectus Excavatum. Ann Noninvasive Electrocardiol.

[11] Ozeke O, Selcuk MT, Topaloglu S, Maden O, Aras D (2006). Brugada-Like Early Repolarisation Pattern Associated with Acute Pericarditis. Emerg Med J.

[12] Sieira J, Ciconte G, Conte G, Chierchia GB, Asmundis C, Baltogiannis G (2015). Asymptomatic Brugada Syndrome: Clinical Characterization and Long-Term Prognosis. Circ Arrhythm Electrophysiol.

