# Supplementary material for: Reflexões sobre as Taxas de Sobrevida em Pacientes com Fenocópia de Brugada
Source: Arq Bras Cardiol. 2025 Oct 28;122(9):e20250330. [Article in Portuguese] doi: 10.36660/abc.20250330 (PMC12671598; doi:10.36660/abc.20250330)
Supplement: Translation

# Translation: Reflections on Survival Rates in Patients with Brugada Phenocopy

**DOI:** 10.36660/abc.20250330i

**Authors:** Gabriel Scarpioni Barbosa, José Nunes de Alencar, Adrian Baranchuk

**Affiliations:** 1 São Paulo SP Brasil Divisão de Medicina – Faculdade Santa Marcelina, São Paulo, SP – Brasil; 2 São Paulo SP Brasil Divisão de Pesquisa – Instituto Dante Pazzanese de Cardiologia, São Paulo, SP – Brasil; 3 Ontário Canadá Divisão de Cardiologia – Kingston Health Science Centre, Queen's University, Ontário – Canadá

**Keywords:** Brugada Syndrome, Survival Rate, Electrocardiography

We read with great interest the article "Survival in Patients with Brugada Phenocopy. Case Series" by Fonseca et al.^1^ The authors describe a case series of patients with Brugada Phenocopy (BrP) and report in-hospital survival. We commend the authors for addressing an important topic related to BrP; however, we would like to comment on methodological considerations and propose recommendations that may strengthen interpretation and guide future BrP reports.

BrP refers to clinical conditions that lead to Brugada-ECG pattern (Br-ECGp) without the genetic substrate of Brugada Syndrome (BS) and an underlying condition that justifies the ECG change.^2^ The term was first used to describe the Br-ECGp caused by the propofol infusion syndrome; however, after several corrections to the concept, drugs that do block the sodium channels were no longer considered part of the BrP spectrum.^3^ BrP represents a diagnostic challenge even for experts. The mean accuracy to identify BrP based only on the 12-lead ECGs was 43±33% among 10 international experts.^4^ Therefore, the clinical context is essential to differentiate BrP from BS. Anselm et al.^5^ presented a systematic diagnostic criterion (Table 1) for the diagnosis of BrP. Emphasis was placed on the recognition of the underlying clinical condition and the low pretest probability for BrP, in addition to the resolution of the Br-ECGp immediately after resolving the underlying condition. Based on the limited description provided by Fonseca et al.,^1^ we cannot exclude the possibility that some cases represented concealed BS unmasked by hyperthermia or other triggers. This misclassification bias could alone skew the observed mortality.

**Table 1:** Systematic diagnostic criteria for Brugada Phenocopy

I) ECG pattern has type 1 or type 2 Brugada morphologic characteristics. (Mandatory).
II) Patient has an underlying condition that is identifiable. (Mandatory).
III) ECG pattern resolves after resolution of the underlying condition. (Mandatory).
IV) There is a low clinical pretest probability of true Brugada syndrome determined by lack of symptoms, medical history, and family history. (Mandatory).
V) Negative results on provocative testing with sodium channel blockers such as ajmaline, flecainide, or procainamide.
VI) Provocative testing is not mandatory if surgical right ventricular outflow tract manipulation has occurred within the last 96 h.
VII) Results of genetic testing are negative (desirable but not mandatory because the SCN5A mutation is identified in only 20%-30% of probands affected by true Brugada syndrome).

The causes of BrP are diverse, and the knowledge about these associations is important to the correct diagnosis. The Br-ECGp was associated with vascular diseases (occlusion myocardial infarction (OMI),^6^ and acute pulmonary embolism,^7^ hydroelectrolytic disorders (hypokalemia^8^ and hyperkalemia,^9^ anatomical variations (pectus excavatum,^10^ and other clinical conditions (septic shock^1^ and acute pericarditis.^11^

Given that BrP lacks the pathologic substrate of BS, its mortality inherently reflects the severity of the underlying condition. BS has a well-established evolution mechanism and survival rate. Patients with BS are prone to ventricular arrhythmias, which may lead to sudden cardiac death. Reported mortality is approximately 1.7% over a mean follow-up period of 73.2±58.9 months.^12^ Fonseca et al.^1^ report an in-hospital survival rate of 26.8%, but this figure is heavily influenced by the predominance of severe conditions in the cohort, such as OMI, acute pulmonary embolism, and septic shock. Had this series included outpatients or milder presentations, survival estimates would likely be substantially higher, reflecting referral bias.

To more precisely assess BrP prognosis, future investigations should consider differential diagnosis, including BrS itself, and enroll larger, more heterogeneous cohorts and compare outcomes in patients with identical underlying conditions presenting with Br-ECGp versus those with typical ECG patterns (for example, OMI with Br-ECGp versus OMI with conventional ST-elevation).

We acknowledge that Fonseca et al.^1^ case series contributes valuable preliminary data on BrP prognosis. Nevertheless, a critical appraisal of potential biases—including referral, misclassification, and confounding—is essential to delineate the true applicability and generalizability of the findings.
