# Supplementary material for: Reflexões sobre as Taxas de Sobrevida em Pacientes com Fenocópia de Brugada
Source: Arq Bras Cardiol. 2025 Oct 28;122(9):e20250330. [Article in Portuguese] doi: 10.36660/abc.20250330 (PMC12671598; doi:10.36660/abc.20250330)
Supplement: Related texts

# Reply: Carta-resposta

**Authors:** De la Torre Fonseca Luis Mariano

**Affiliations:** 1 Faculdade Santa Marcelina Divisão de Medicina São Paulo SP Brasil Divisão de Medicina – Faculdade Santa Marcelina, São Paulo, SP – Brasil

Agradecemos sinceramente aos autores pelo interesse em nosso artigo "Sobrevida em Pacientes com Fenócopia de Brugada. Série de Casos", publicado recentemente nos Arquivos Brasileiros de Cardiologia.^1^ Valorizamos particularmente os insights metodológicos e conceituais fornecidos, que contribuem significativamente para a discussão científica em torno da fenocópia de Brugada (FB), uma entidade clínica ainda em fase de definição e padronização.

Concordamos plenamente que a FB representa um desafio diagnóstico, mesmo para eletrofisiologistas experientes, devido à sua sobreposição fenotípica com a síndrome de Brugada (SB) e à necessidade de excluir rigorosamente condições subjacentes que possam mimetizar o padrão eletrocardiográfico de Brugada (PEBr). Em nossa série de casos, os critérios diagnósticos atualmente aceitos foram rigorosamente aplicados, incluindo a resolução do PEBr após o tratamento da condição subjacente, bem como a contextualização clínica completa de cada caso. Conforme destacado em sua carta, esses elementos são essenciais para diferenciar FB da SB, uma vez que o diagnóstico se baseia não apenas nos achados do ECG, mas também no cenário clínico e em sua progressão temporal.

Em relação ao importante ponto levantado sobre o potencial viés de classificação, gostaríamos de esclarecer que todos os casos incluídos em nossa série foram revisados e discutidos por um painel de especialistas em cardiologia clínica e eletrofisiologia. Causas secundárias capazes de mimetizar o PEBr — como hipertermia, distúrbios eletrolíticos, exposição a bloqueadores dos canais de sódio e outros gatilhos bem documentados — foram sistematicamente descartadas. No entanto, reconhecemos que o teste de provocação farmacológica com ajmalina ou flecainida não foi realizado devido à instabilidade clínica dos pacientes e ao risco potencial de induzir arritmias ventriculares ou agravar o comprometimento hemodinâmico. Essa limitação, que foi claramente declarada em nossa seção de métodos, representa um dilema ético e clínico frequentemente encontrado na avaliação de pacientes críticos.

Quanto à taxa de sobrevida hospitalar relatada (26,8%), concordamos que ela reflete mais a gravidade das condições subjacentes (por exemplo, infarto agudo do miocárdio com oclusão, embolia pulmonar aguda, choque séptico) do que o próprio PEBr. De fato, uma coorte com maior heterogeneidade clínica — incluindo casos ambulatoriais ou apresentações mais leves, como pericardite aguda ou pectus excavatum — provavelmente poderia ter apresentado estimativas de sobrevida mais elevadas. Apreciamos essa observação, pois ressalta a relevância do viés de encaminhamento, que deve ser cuidadosamente considerado na interpretação dos resultados de qualquer série de casos.

Por fim, concordamos plenamente com a necessidade de estudos futuros envolvendo populações maiores e mais diversas, com comparações controladas entre pacientes com condições subjacentes idênticas, com e sem PEBr. Somente por meio de estudos multicêntricos e prospectivos será possível determinar se a presença do padrão de Brugada nesses contextos traz implicações prognósticas independentes ou se representa simplesmente um epifenômeno.

Agradecemos novamente aos autores pela leitura cuidadosa e avaliação crítica do nosso trabalho e compartilhamos o objetivo comum de avançar na compreensão desta entidade clínica complexa e desafiadora.

**Fonseca et al**

## References

[1] Fonseca LMT, Cedeño RA, Bello LAH, Díaz-Heredia J, Quezada DMP, Castro RAG (2025). Survival in Patients with Brugada Phenocopy. Case Series. Arq Bras Cardiol.

# Reply

**Authors:** De la Torre Fonseca Luis Mariano

**Affiliations:** 1 São Paulo SP Brasil Divisão de Medicina – Faculdade Santa Marcelina, São Paulo, SP – Brasil

We sincerely thank the authors for their interest in our article, "Survival in Patients with Brugada Phenocopy. Case Series," recently published in *Arquivos Brasileiros de Cardiologia*.^1^ We particularly value the methodological and conceptual insights provided, which meaningfully contribute to the scientific discussion surrounding Brugada phenocopy (BrP), a clinical entity still undergoing definition and standardization.

We fully agree that BrP presents a diagnostic challenge, even for experienced electrophysiologists, due to its phenotypic overlap with Brugada syndrome (BS) and the necessity of rigorously excluding underlying conditions that may mimic the Brugada electrocardiographic pattern (Br-ECGp). In our case series, currently accepted diagnostic criteria were rigorously applied, including the resolution of the Br-ECGp following treatment of the underlying condition, as well as thorough clinical contextualization of each case. As highlighted in your letter, these elements are essential to differentiate BrP from BS, since the diagnosis relies not only on ECG findings but also on the clinical scenario and its temporal progression.

Regarding the important point raised about potential classification bias, we would like to clarify that all cases included in our series were reviewed and discussed by a panel of experts in clinical cardiology and electrophysiology. Secondary causes capable of mimicking Br-ECGp—such as hyperthermia, electrolyte disturbances, exposure to sodium channel blockers, and other well-documented triggers—were systematically ruled out. However, we acknowledge that pharmacological provocation testing with ajmaline or flecainide was not performed due to the clinical instability of the patients and the potential risk of inducing ventricular arrhythmias or worsening hemodynamic compromise. This limitation, which was clearly stated in our methods section, represents an ethical and clinical dilemma often encountered when evaluating critically ill patients.

As for the reported in-hospital survival rate (26.8%), we agree that it reflects more the severity of the underlying conditions (e.g., acute myocardial infarction with occlusion, acute pulmonary embolism, septic shock) than the Br-ECGp itself. Indeed, a cohort with greater clinical heterogeneity—including outpatient cases or milder presentations such as acute pericarditis or pectus excavatum—could likely have shown higher survival estimates. We appreciate this observation, as it underscores the relevance of referral bias, which must be carefully considered when interpreting results from any case series.

Lastly, we fully agree on the need for future studies involving larger and more diverse populations, with controlled comparisons between patients with identical underlying conditions, with and without Br-ECGp. Only through multicenter, prospective studies will it be possible to determine whether the presence of the Brugada pattern in these contexts carries independent prognostic implications or simply represents an epiphenomenon.

We again thank the authors for their thoughtful reading and critical appraisal of our work, and we share the common goal of advancing the understanding of this complex and challenging clinical entity.

**Fonseca et al**
